# Emerging Functions for snoRNAs and snoRNA-Derived Fragments

**DOI:** 10.3390/ijms221910193

**Published:** 2021-09-22

**Authors:** Maliha Wajahat, Cameron Peter Bracken, Ayla Orang

**Affiliations:** 1 School of Biological Sciences, Faculty of Sciences, University of Adelaide, Adelaide, SA 5000, Australia; malihawajahat@student.adelaide.edu.au (M.W.); cameron.bracken@unisa.edu.au (C.P.B.); 2 Centre for Cancer Biology, An Alliance of SA Pathology and University of South Australia, Adelaide, SA 5000, Australia; 3 School of Medicine, Discipline of Medicine, University of Adelaide, Adelaide, SA 5000, Australia

**Keywords:** non-coding RNAs, snoRNAs, sdRNAs, microRNAs, tRNAs

## Abstract

The widespread implementation of mass sequencing has revealed a diverse landscape of small RNAs derived from larger precursors. Whilst many of these are likely to be byproducts of degradation, there are nevertheless metabolically stable fragments derived from tRNAs, rRNAs, snoRNAs, and other non-coding RNA, with a number of examples of the production of such fragments being conserved across species. Coupled with specific interactions to RNA-binding proteins and a growing number of experimentally reported examples suggesting function, a case is emerging whereby the biological significance of small non-coding RNAs extends far beyond miRNAs and piRNAs. Related to this, a similarly complex picture is emerging of non-canonical roles for the non-coding precursors, such as for snoRNAs that are also implicated in such areas as the silencing of gene expression and the regulation of alternative splicing. This is in addition to a body of literature describing snoRNAs as an additional source of miRNA-like regulators. This review seeks to highlight emerging roles for such non-coding RNA, focusing specifically on “new” roles for snoRNAs and the small fragments derived from them.

## 1. Introduction

Since discovering the functions of structural non-coding RNAs (ncRNAs) such as rRNAs and tRNAs in the 1950s, it has been apparent that RNAs do not simply serve as intermediaries in the direction of protein production [1,2,3,4,5]. Advancing sequencing technologies have greatly expanded our understanding of the classes of ncRNAs and many of their roles [6,7], though given more than 95% of the genome is transcribed, the functional significance of many remains unknown [8,9,10]. ncRNAs can be classified into two groups: housekeeping or structural RNAs such as tRNAs, snoRNAs, and rRNAs; and regulatory RNAs such as miRNAs, siRNAs, and piRNAs [11,12]. These distinctions however are not always clear. Long ncRNAs (lncRNAs) and promoter associated RNAs (paRNAs) for example may fall under either category depending upon definition, as both serve structural functions that have regulatory implications [13,14]. Longer structural RNAs can also be processed within cells, generating smaller fragments that exert essential regulatory roles that, at least in some cases, are conserved across species [15,16,17,18,19,20].

Given the plethora of transcripts and degradation products within cells, assigning function to products of RNA processing is not simple. However, there is a growing body of evidence to demonstrate functional significance of smaller RNAs that are derived from larger non-coding precursors, and it is clear that in many cases one cannot dismiss the potential for these small RNAs to regulate biological processes. tRNAs are one such example, considered for decades to only be responsible for facilitating the addition of amino acids to growing polypeptide chains, but now known to generate tRNA-derived fragments that perform essential gene regulatory functions such as gene silencing, epigenetic inheritance, and ribosome biogenesis [21,22,23,24]. Similarly, snoRNAs were previously known to direct the chemical modification of rRNAs, but a far more complex picture is also emerging, with snoRNA-derived fragments (sdRNAs) regulating gene expression and being linked to the development of various pathologies, including cancer [25,26,27,28]. As new technologies and methodologies continue to be developed, the number of ncRNAs and the diversity of their functions will only continue to grow. This review primarily focuses on emerging roles for snoRNAs and snoRNA-derived fragments, looking at their mechanisms of action, association with pathologies, and potential as biomarkers.

## 2. SnoRNA Classes and Canonical Function

Small nucleolar RNAs (snoRNAs) were discovered in mammalian cells in 1960, where their localization suggested a possible link to ribosomal RNAs (rRNAs), another class of ncRNA discovered several decades prior [5,29]. It was discovered that snoRNAs are primarily responsible for post-transcriptional modifications, directing 2′-O-ribose methylation and psuedouridylation of rRNAs and snRNAs (small nuclear RNAs) to enhance ligand interactions and guide three-dimensional folding, thereby fine-tuning ribosome and spliceosome function [30,31]. On their own, snoRNAs cannot bring about base modification and isomerization of rRNA. Instead, snoRNAs act as guide RNAs, associating with a specific set of proteins to form snoRNA–ribonucleoprotein complexes (snoRNPs) that protect snoRNAs from exonucleolytic degradation [32] and direct methyltransferase and pseudouridine synthase enzymes to the modification site via complementary base pairing with the target rRNA [29,33,34,35].

SnoRNAs represent an ancient genetic lineage, present in both Archaryotes and Eukaryotes, which maintain an extraordinary degree of sequence conservation within the structural motifs by which snoRNAs are classified [36]. The two main classes of snoRNAs comprise C/D box snoRNAs (also known as SNORDs) and H/ACA snoRNAs (also known as SNORAs) that respectively guide the methylation and psuedouridylation of their targets. A third class, small Cajal body specific RNAs (scaRNAs), possess composite features of the other two classes.

**Figure 1:**
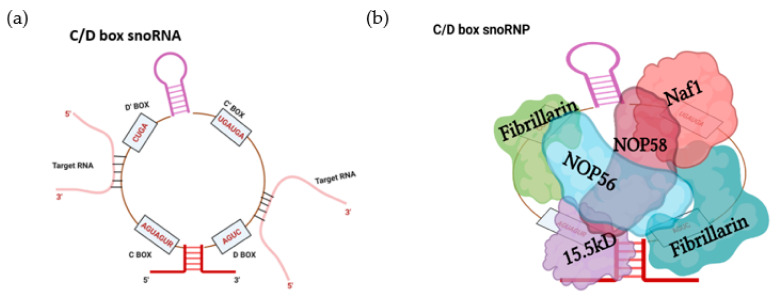
The C/D box snoRNA structural features and associated protein cohort: (**a**) Stem-bulge-stem (K-turn) structure is a characteristic feature of the C/D box snoRNAs. At the 5′ and 3′ termini lie the Box C (RUGAUGA) and Box D (CUGA) conserved sequences whilst the boxes C′ and D′ represent internal less-conserved sequence elements. The guide sequences (antisense elements) upstream of the box D/D′ are complementary to target RNAs and form the sequence recognition component. (**b**) Fibrillarin (green), Nop58 (violet), Nop56 (Blue), and 15.8kD (Purple) proteins associate with C/D box snoRNAs to form a functional snoRNP. Fibrillarin enzymatically facilitates 2′-O-ribose-methylation of target RNAs while the other three proteins facilitate the localization, maturation, and stability of C/D box snoRNAs.

The first class of snoRNA, C/D box snoRNAs, are characterized by the presence of two conserved sequence motifs, box C (RUGAUGA) and box D (CUGA), near the 5′ and 3′ termini, respectively [29,34,37]. The base pairing between the 5′ and 3′ ends brings these two boxes in close proximity, forming a terminal stem structure harboring a specific kink-turn [29,34,37,38]. Additionally, these snoRNAs also possess C′ and D′ boxes—less conserved counterparts of C and D boxes [34,39]. Intramolecular base pairing forms structures that enable SNORDs to serve as a scaffold for the assembly of the proteins (NOP56, NOP58, 15.5K, and fibrillarin) that are essential for stability, localization, and function [29,40,41,42]. SNORDs recognize the target RNAs via a short 10–21 nucleotide long guide region (also known as antisense elements; ASE), situated upstream of the D or D′ boxes [29,32,43]. Fibrillarin, a methyltransferase that executes the function of the snoRNP, then modifies the fifth nucleotide upstream of the D or D′ box motif [29,35] (Figure 1).

**Figure 2:**
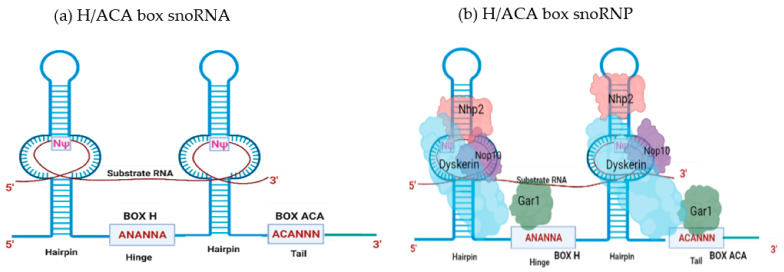
The H/ACA snoRNA structural features and associated protein cohort: (**a**) The H/ACA snoRNA family is characterized by two hairpins separated by the hinge region. H/ACA comprises box H and box ACA, the two conserved motifs, which are located in the hinge region and the 3′ end, respectively. The psuedouridylation pocket is present in each hairpin of the H/ACA snoRNA that forms the interaction interface for target RNAs (shown in red) and is the site of psuedouridylation (NΨ) of the substrate RNA. (**b**) The proteins DKC1(cyan), Nop10(violet), Gar1 (Green), and Nhp2 (pink) associate with H/ACA snoRNA to form the H/ACA SnoRNP complex. DKC1 (the pseudouridine synthase dyskerin) directly promotes psuedouridylation. Additionally, Nop10 and Nhp2 have been demonstrated to be critical for H/ACA stability and function. Moreover, GAR1 has been shown to enhance the catalytic activity of dyskerin and to facilitate the release of substrate RNA.

The second class of snoRNA, H/ACA snoRNAs, are characterized by the presence of a 3′ (ACA) tail and two hairpin structures connected by a hinge region bearing a conserved (ANANNA) motif (also known as the H motif, BOX H) [29,34,39,44]. Each hairpin contains an internal loop that forms the psuedouridylation pocket as these regions harbor complementarity to the target RNAs [29,44]. The uridine residue in the target RNA, 14–15 nucleotides upstream of the H and/or ACA box, is then usually psuedouridylated by dyskerin, a pseudouridine synthase that is a component of the SNORA-associated snRNP (also containing NHp2, NOP10, and GAR1) [39,45,46] (Figure 2).

The third subfamily of snoRNAs, scaRNAs, are localized in sub-nuclear regions known as Cajal bodies on account of an additional Cajal body localization motif (UGAG) [47]. scaRNAs aid the formation and maturation of spliceosomes and ribosomes, respectively, by methylation and psuedouridylation of small nuclear RNAs (snRNAs) and rRNAs [39,47,48].

All three families of snoRNA not only fine-tune the ribosomes and spliceosomes by expanding the chemical repertoire of RNA to facilitate RNA–protein and RNA–RNA interactions but also protect the target RNAs from nucleolytic degradation [29,39,47]. While most of these snoRNAs are not essential for cell viability, as the modifications exerted by them are responsible for fine-tuning translation and splicing, SNORD3 (U3), SNORD118, SNORD14, and SNORA73 are essential for cell viability on account of their direct roles in pre-rRNA cleavage [5,29,49,50]. These differ from other snoRNAs in their processing and associated protein cohorts [5]. However, these snoRNAs have been the focus of other reviews [51,52,53] and will not be discussed further here.

### Biogenesis

**Figure 3:**
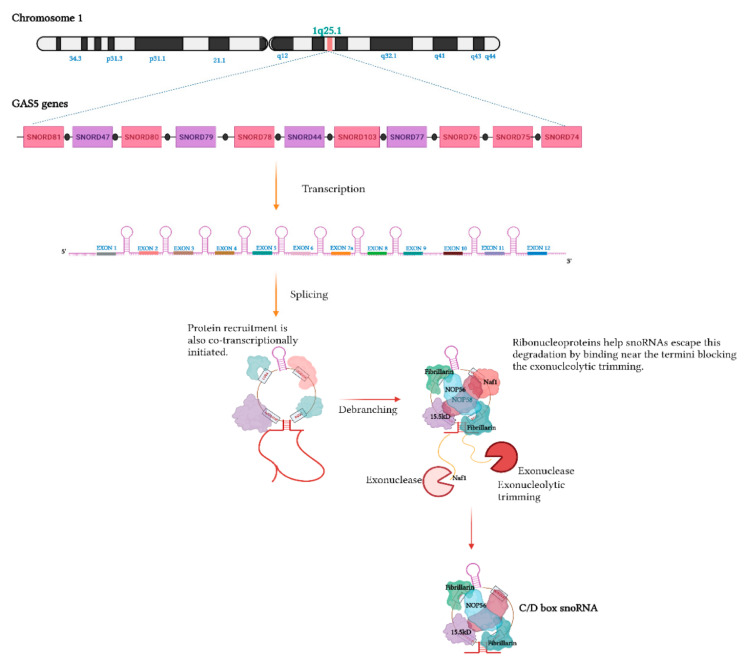
Encoding of multiple snoRNAs within a single non-coding transcript: Eleven intronically derived C/D box snoRNAs are produced from the growth arrest-specific 5 gene, *GAS5*. snoRNP assembly is initiated co-transcriptionally with snoRNP-associated proteins (15.5 kD, fibrillarin, NOP58, and NOP56) and auxiliary factors (Naf1, Shq1), protecting the snoRNAs from exonuclease trimming. The GAS5 transcript contains snoRNAs separated by exons; these exons in GAS5 do not code for any protein 64. This transcription style in humans is different than the polycistronic transcription observed in yeast 65. Additionally, the open reading frame for GAS5 transcript, not being required for protein production, is poorly conserved across species. However, a striking conservation exists across species for intronically encoded snoRNAs. Taken together, this suggests biologically significant roles for *GAS5*-encoded snoRNAs.

Typically, snoRNAs are transcribed from introns, usually embedded in genes that code for proteins related to ribosome biogenesis and function, thus providing a mechanism that ensures that ribosomes and associated snoRNAs are present in the correct proportions [54,55]. Other snoRNAs are transcribed from the introns of non-coding genes, such as *GAS5* and *SNHG1*, from which eleven and nine snoRNAs are respectively derived [54,56,57] (Figure 3).

**Figure 4:**
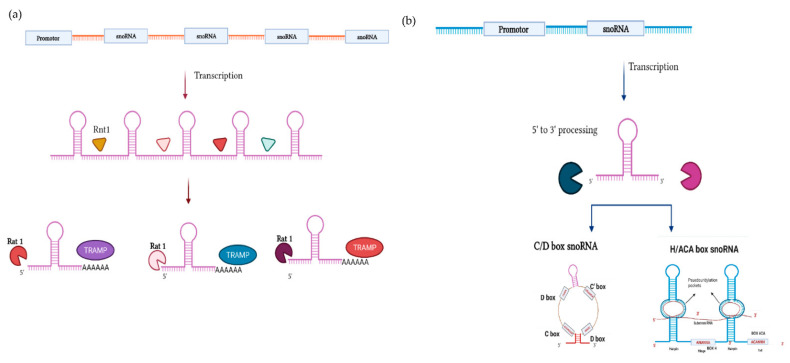
Production of snoRNAs from polycistronic transcriptions and from independently transcribed genes: (**a**) Only a few snoRNAs in yeast are transcribed polycistronically—an example of which are the seven C/D box snoRNA transcribed polycistronically from the cluster located on chromosome 8. The snoRNAs are transcribed as a single transcript from an independent promotor with no intervening exons present in between. The snoRNAs are processed by the nucleases Rnt1 and Rat1. Rnt1P facilitates the removal of m7G cap and cleaves the transcript at Rnt1 cleavage sites such that an entry site for the exonucleases is created for the further processing of pre-snoRNAs. Almost all SNORDs are synthesized having capped 5′ extensions; however, the uncapped 5′ extensions are processed by Rat1p and Xrn1p. Additionally, the 3′ processing is performed by TRAMP4/5 and Rrp6 [63,64]. Rnt1-dependent 5′ processing of snoRNAs is critical for its function [65]. (**b**) Some of the snoRNAs in yeast are transcribed monocistronically, having an independent promotor. This type of snoRNA is released from the intergenic parent via endonucleolytic cleavage followed by processing by 5′ to 3′ exosomes.

The snoRNAs that are intronically encoded evade exonucleolytic attack via the co-transcriptional binding of ribonucleoproteins that block exonucleolytic trimming [58,59]. The RNP components previously mentioned, along with some auxiliary factors (notably, Naf1, Shq1, and NUFLP), are critical for processing stability and nucleolar localization [34]. snoRNPs are then transported to Cajal bodies for additional maturation and processing prior to their delivery to nucleoli, where they exert their ncRNA-modification roles [59,60]. Furthermore, typically in plants and yeast, some of the snoRNAs are transcribed polycistronically (Figure 4a), which is different from the transcription of *GAS5*, where snoRNAs are separated by exons [61,62]. Additionally, other snoRNAs in yeast are transcribed autonomously by polymerase II [10,34] (Figure 4b).

## 3. Diversification of ncRNA Function

As will be discussed, the view of snoRNA function held over decades is being reconsidered in light of novel technologies and methodologies that reveal more diverse roles [39]. This will be the focus of the remainder of the review, though it is worth bearing in mind a similar re-evaluation of roles is also being made for other classes of ncRNA [66,67,68,69], ultimately speaking to the complexity through which biological systems are regulated. Key examples of these will be discussed.

### 3.1. miRNAs

In many regards, parallels can be drawn between miRNAs and snoRNAs in that they are both processed from longer RNAs that may either be devoted to their specific production or may be located within the introns of protein coding genes. Both miRNAs and snoRNAs also serve as target recognition components of larger ncRNA–protein complexes, bringing associated proteins to their target transcripts in order to exert a biological effect. In the case of miRNAs, this effect is the post-transcriptional silencing of target transcripts, either by destabilizing the mRNA target or suppressing its translation, mediated by Argonaute (AGO) and other members of the RNA-induced silencing complex (RISC) of which the miRNA is the targeting component [70].

The canonical miRNA biogenesis pathway has been established for some time, involving two enzymatic reactions catalyzed respectively within the nucleus and cytoplasm by the Drosha and Dicer enzymes, each functioning within multi-component protein complexes [70,71]. Additional DROSHA- and DICER-independent mechanisms however have also been demonstrated to generate functional miRNAs [17,25,72,73,74,75]. Similarly, although the canonical, post-transcriptional mechanisms by which miRNAs silence gene expression in the cytoplasm have been established over the past two decades, new evidence again suggests this represents an incomplete picture of function.

This is particularly apparent with the nucleus, where cell fractionation has revealed the presence of abundant mature, fully processed miRNAs [76] and the existence of a smaller AGO-containing miRNA–RNP complex known as RNA-induced transcriptional silencing complex (RITS) [77,78,79]. Within nuclei, miRNAs are purported to play direct roles in the regulation of transcription, either in a suppressive (transcriptional gene silencing, TGS) or activating (transcriptional gene activation, TGA) manner. In the context of TGS, the recruitment of miRNAs to target promoters (either directly or via a promoter-associated RNA intermediary) can lead to the suppression of gene activity through the recruitment of repressive factors, such as EZH2 leading to hyper-chromatinization [80,81]. Similarly, TGA can recruit positive epigenetic regulators [82], or may function indirectly by binding and repressing other ncRNAs that themselves play inhibitory roles at the promoters to which they are targeted [83,84]. Alternately, nuclear miRNAs might exert direct regulatory functions in alternative splicing, as implied by altered splicing patterns in response to the knockdown of key miRNA-pathway genes (AGO, DROSHA, DICER) and by the association of these proteins with known splicing factors [85,86].

### 3.2. tRNAs and tRFs

In close parallel to snoRNAs, for decades tRNAs also had a largely single and well-defined role—in this case, mediating the transport of amino acids during translation to the growing polypeptide chain [87]. Also, in parallel to snoRNAs, central to this function is their structure, with internal base pairing, modulated by site-specific post-transcriptional modification, creating a classic L-shaped fold comprising three arms: the anticodon loop, which binds to the target amino acid and the T, and D arms that interact with each other to stabilize the overall three-dimensional structure [88]. tRNA processing during biogenesis is mediated via the RNAses P and Z that facilitate removal of the 5′ leader and 3′ trailer sequences, respectively [20], whilst tRNA removal is controlled by rapid tRNA decay (RTD) and nuclear surveillance and degradation of hypomodified pre-tRNA pathways, respectively [89,90].

Interestingly, and of direct relevance to this review, next-generation sequencing has also revealed the generation of smaller tRNA-derived fragments (tRFs) [91]. Though initially dismissed as inconsequential degradation products, comparative analysis revealed that tRFs were not just the random products of degradation but were instead evolutionarily conserved across species and present in all eukaryotes [92,93,94,95]. Thereafter, a myriad of studies aimed at understanding the functional significance of these tRFs revealed that dysregulation of these fragments is associated with a number of pathologies, including cancer and neurodegenerative disorders [95,96,97].

tRFs broadly fall into four classes, depending on their size and the region from which they are derived [98]. tRF-1 members are derived from the 3′trailer regions of the pre-tRNAs, while 5′-tRFs (14–30nt long) and 3′-tRFs (18 or 22nt long) are generated from the cleavage of 5′ and 3′ regions of mature tRNAs, respectively [99]. The enzymes involved in the generation of these fragments are yet to be discovered [100]. However, specific cutting between A/U nucleotides that is observed suggests the involvement of endonucleases such as PhyM or U [101]. The remaining tRF class are generated during stress conditions (such as hypoxia and starvation) by angiogenin (ANG)-mediated cleavage of the anticodon loop. These stress-induced tRNA fragments (tiRNAs) themselves comprise multiple subclasses: 5′-tiRFs, 3′-tiRFs (31–40 nt long) [98,102], and tRF-2 generated during hypoxia that do not possess classic 3′ and 5′ end functional groups [103].

Different tRF sizes and termini suggest different processing mechanisms [104], whilst different localization of the fragments (tRF-1 and 3 are primarily cytoplasmic, while tRF-5 is nuclear [99]) suggest different functions. Though many of these functions remain a mystery, a growing body of research now reports important roles for tRFs. Gene regulatory functions of tRFs were first suggested by immunoprecipitation studies that reported the association of tRFs with Argonaute 1, 3, and 4 (but not with AGO2) [99], which is anatomically similar to that of the miRNA-AGO-target complex. It does not appear that these tRFs silence transcripts in the same manner as miRNAs, but rather sequester the AGO proteins and thereby prevent miRNAs from exerting their silencing functions [104,105].

Further indicating tRF functionality was the observation that knockdown of tRF-Gly-GCC (tRF-GG) represses genes that are associated with the expression of endogenous retroviral element (ERVL) [106]. Although no association between tRF-GG and ERVL was found, research instead identified that tRF-GG modulates the expression of the U7 snRNA, which ultimately affects ERVL expression via the regulation of histone pre-mRNA 3′ processing [107]. Similarly, tRFs affects the Cajal body biogenesis and hence ultimately regulates the generation of other ncRNAs, including snoRNAs and snRNAs [107].

Additionally, there are literally hundreds of studies purporting that dysregulation of tRFs is related to cancers of various types [108,109,110]; however, the underlying mechanisms remain largely unknown, both in terms of what alters tRNA processing and what signaling cascades are triggered by tRF dysregulation [20]. In addition to their potential utility as biomarkers, tRF levels might also indicate how well cells will respond to various kinds of therapy, though again mechanistic explanations remain poorly understood [103,111].

### 3.3. SnoRNAs—Mechanistic Roles in Cancer and Their Capacity as Biomarkers

One of the first indications that snoRNAs could play important roles beyond those for which they are traditionally known came with the initial observation that the human snoRNA h5sn2 is downregulated in meningiomas compared with normal brain tissue [112]. Thereafter, a myriad of studies emerged associating snoRNA dysregulation with cancer progression [34]. Some of these were linked to the function of the p53 tumor suppressor. For example, the upregulation of SNORA18L5 in hepatocellular carcinoma (HCC) led to hyperactive ribosome biogenesis, increasing levels of mature rRNAs that in turn caused the accumulation of the ribosomal proteins RPL5 and RPL11 in the nucleolus. This prevented the binding and sequestration of MDM2 by these ribosomal proteins, which in turn promoted cancer via the subsequent ubiquitylation and degradation of p53 [112,113]. The p53 pathway is also engaged upon the knockdown of the U3 and U8 snoRNAs, with a potent p53 anti-tumor surveillance response occurring after the blockage of ribosome synthesis that results from U3 and U8 no longer being available for pre-rRNA processing [114]. Acting as a transcription factor, p53 is also directly associated with snoRNA expression, such as in colorectal carcinomas (CRCs), where p53 controls the expression of *GAS5*, a host gene for multiple snoRNAs that influence apoptosis and the cell cycle [26]. Similarly, via the regulation of the Ets2 transcription factor, p53 controls a host of snoRNA genes in a model of osteosarcoma. Accordingly, deletion of Ets2 in p53-mutant mice resulted in the downregulation of these snoRNAs and the reversal of the pro-metastatic phenotype that the p53 mutation otherwise promotes [115].

Several studies have also ascribed roles for snoRNAs within other cancer-associated signaling pathways, such as PI3K/AKT, which plays a pivotal role in cell differentiation, proliferation, and survival [116]. Though the precise mechanisms are yet to be discovered, two snoRNAs that are upregulated in hepatocellular carcinomas (HCCs), SNORA11 and SNORD126, both increase PI3K/AKT signaling, with SNORA11 knockdown inducing cell cycle arrest and suppressing the proliferation, migration, and invasion of cell lines and decreasing HCC growth in an animal model [117,118].

Cancer stem cells present challenges to the elimination of cancers on account of their capacity to self-renew, differentiate, and seed tumors [119]. Increasing evidence suggests intricate roles for snoRNAs in cancer stem cells [34]. Aldehyde dehydrogenase (ALDH) is a cancer stem cell marker, and it has been reported that the ALDH-positive cancer cells manifest dysregulation of approximately 22 snoRNAs compared with ALDH-negative cells in non-small lung cancer (NSCLC) [120]. Among these, upregulation of SNORA3 and SNORA43 was associated with poor survival, with SNORA42 knockdown reducing the self-renewal of cancer cells with stem-like properties [120]. SNORD78 was also shown to be crucial for the self-renewing ability of cancer stem cells in NSCLCs [121]. However, the events that trigger the dysregulation of snoRNAs and most of their consequent effects remain largely unknown [34].

As demonstrated by the examples above, the dysregulation of snoRNA expression is prevalent in various cancers [34]. Coupled with their stable expression allowing detection in blood, plasma, urine, and other body fluids [34], snoRNAs are therefore potential candidates as cancer biomarkers [122]. The upregulation of HBI-239 for example is a prognostic marker of angioimmunoblastic T-cell lymphoma [123]. The overexpression of SNORA21, a critical oncogene in CRC, is also related to poor patient survival and can be used as a prognostic marker [124], whilst SNORD114–10 can be used as a pre-diagnostic biomarker in ovarian cancer, as its downregulation correlates with metastasis [125]. Additionally, snoRNA differential expression profiles can facilitate subgroup classification of leukemia [126]. For additional discussion of the potential of snoRNAs in cancer and as biomarkers, the reader is directed to a number of reviews [127,128,129,130,131,132,133].

## 4. Emerging snoRNA Non-Canonical Functions

Though historically snoRNAs are known to facilitate psuedouridylation and methylation of rRNA and snRNA, emerging studies suggest that additional types of modifications, or different RNA targets for modification, also exist [39]. For example, the acetylation of two N4 cytidine residues in 18S rRNA is brought about by the orphan snoRNAs snR4 and snR45, which guide the cytidine acetyltransferase Kre33 to the target location [134,135]. These two N4 acetylcytidine residues in 18S are located within the peptidyl transferase and decoding region on helix 34 and 45, respectively [134,136], with the loss of rRNA modifications in these two regions resulting in impaired translation [137,138]. Hence, acetylation facilitated by snR4 and snR45 are deemed critical for ensuring translational fidelity [134,136,137,139]. Orphan snoRNAs do not have any ostensible rRNA, or any other target RNA they interact with [140].

Post-transcriptional modification of tRNAs are also prevalent at the anticodon wobble position and at the position 3′ to the anticodon triplet that stabilizes the mRNA–tRNA interaction [134]. The cytidine that is present at the wobble region is methylated, and it has been shown that SNORD97, which possesses sequence complementary to the anticodon loop of elongator tRNAMet, helps direct this process. [141,142]. Methylation of the tRNA at this position is protective against cleavage by stress-induced endonucleases such as angiogenin [143]. Although it has taken more recent work to elucidate such examples, earlier co-immunoprecipitation studies did observe physical snoRNA–tRNA interactions, and it is likely further snoRNA–tRNA interactions remain to be uncovered [144].

In addition to non-coding RNAs, high-throughput sequencing of ligated RNAs also reveals interactions between snoRNAs and mRNAs, such as SNORD83B, an orphan snoRNA that stabilizes the levels of target mRNAs (NOP14, RPS5, and SRSF3). Although the full mechanism remains uncharacterized, direct base pairing between the snoRNA and its target mRNAs is required [145].

snoRNAs have also been implicated in regulating alternative splicing of mRNAs [10], such as that of the serotonin receptor 2c (Htr2c), which is directed by the orphan snoRNA SNORD115 [39]. SNORD115 possesses an 18nt region of complementarity to the alternative exon 5b of the Htr2c mRNA [39]. Alternative splicing results in two receptor isoforms: one with the inclusion of exon 5b, resulting in the long functional variant, and one where exon skipping results in a non-functional form [146]. SNORD115 promotes the generation of the long functional isoform by promoting the inclusion of the alternative exon [147]. Further, SNORD115 competes with Adenosine deaminases acting on RNA (ADAR) enzymes that otherwise catalyze adenosine-to-inosine (A–I) editing within this region [148], resulting in changes to three amino acids within exon 5 that affects coupling to G-proteins and downstream receptor signaling cascades [149]. Five additional alternative splicing targets of SNORD115 were also identified [150], whilst SNORD116 located at the same chromosomal locus was similarly implicated as a splicing regulator, as many of its predicted binding sites are located at alternate splice junctions [151]. SNORD27 also directs splicing of the E2F7 transcription factor [10], and SNORD88C regulates alternate splicing of the fibroblast growth factor receptor (FGFR3) [152], masking cryptic splice sites to prevent exon inclusion [152].

In addition to guiding a more diverse array of post-transcriptional modification or regulating alternative splicing, snoRNAs also participate in the 3′-processing of mRNAs. In an attempt to better understand poly(A) site selection, it was revealed that a subset of snoRNAs is associated with the mammalian 3′-end processing complex, with snoRNAs interacting with Fip1, a component of the cleavage and polyadenylation specificity factor (CPSF) [153]. One of these Fip1-interacting snoRNAs, SNORD50A, inhibited 3′ mRNA processing for a subset of transcripts by blocking the site of Fip1-poly(A) interaction [153]. Additionally, snoRNAs are known to accumulate and function within the nucleolus; however, a recent study revealed the accumulation of SNORD32A, SNORD33, and SNORD35A in the cytosol during stress conditions [28]. This suggests that snoRNAs may also have functions outside the nucleus.

### 4.1. snoRNA-Derived RNAs (sdRNAs)—Function

As is reported with tRNAs, high-throughput sequencing has revealed that snoRNAs are also processed to generate smaller fragments, of which at least a portion do not appear to be simple degradation products. The evidence to support this claim, other than a trickle of reports of functional effects of sdRNAs described later in this section, is that sdRNA expression is often conserved across species [42] and that highly abundant sdRNAs may be derived from weakly expressed snoRNAs [17]. Further, the existence of stable snoRNA-derived fragments but not fragments derived from other regions of the same snoRNA imply specific stabilization.

On the whole, sdRNAs are classifiable into several categories based upon their origin and length: H/ACA box snoRNAs generate 20–24 nt sized fragments mainly derived from the 3′ end [17,25], whereas the C/D box snoRNAs are reported to produce two types of fragments, one greater than 26nt in length and another 17–19 nt mainly derived from the 5′ end [17,42]. sdRNA production is widespread, with more than half of all snoRNAs shown to produce smaller fragments [42]. Whilst the functional significance of many of these are currently unknown, their abundance at least suggests the possibility of widespread novel roles, for which at least some have been reported.

**Figure 5:**
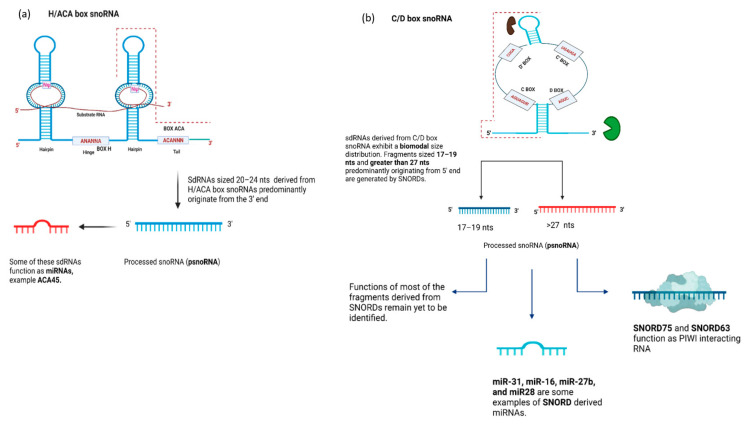
Fragments derived from C/D and H/ACA box snoRNAs: (**a**) The fragments generated by SNORA usually lie in a size range between 20 and 24 nucleotides [17]. There is evidence in the literature that some of these fragments act as miRNAs, an example of which is ACA45, a miRNA derived from H/ACA scaRNA15. ACA45 is around 20–22 nt in length, originating from the 3′ half of the ACA part of the scaRNAs [25]. We know that ACA45 synthesis is DICER-dependent and DROSHA-independent. The processing enzymes for most of these sdRNAs are still unknown [10]. The functions of the other fragments derived from H/ACA snoRNA remain largely unknown [42]. (**b**) Fragments lying in two different size ranges are derived from SNORDs [17]. One group lies in the size range of 17–19 nt while the other group comprises sdRNAs that are greater than 27 nt. Additionally, there are reports that some of these fragments function as miRNAs [18], while some of the fragments act as piRNAs [27]. Nevertheless, the functions and the processing enzymes of most of the SNORD-derived fragments remain unknown [42].

One obvious possible function for sdRNAs is as a novel source of miRNAs, as was initially reported looking at small RNAs co-immunoprecipitating with AGO1 and AGO2 in human embryonic kidney 293 cells (HEK293) [25]. One of the fragments immunoprecipitated with AGO2 was found to be a sdRNA derived from H/ACA scaRNA15 (ACA45) [25] (Figure 5a). Further investigation employing luciferase reporter assays and bioinformatic prediction algorithms revealed that ACA45 is capable of targeting the CDC2L6 mRNA in a miRNA-like manner [20,25]. As with miRNAs produced via their canonical biogenesis pathway, ACA45 processing requires Dicer; however, unlike most miRNAs, processing is independent of Drosha [17]. Several other sdRNAs also function as miRNAs, with their activities again verified using reporter gene assays [18,25,154]. Nevertheless, with additional sequencing, it is likely that the list sdRNAs for which miRNA-like functions are claimed will continue to grow.

SnoRNAs are also a reported source of piRNAs, a class of non-coding RNA 26–31 nt in size that associate with AGO-related PIWI proteins and that are involved in the degradation of transposon RNA and the epigenetic regulation of gene expression [27]. In one study, a number of snoRNA-derived piRNA candidates were identified in primary T lymphocytes, with one particular piRNA derived from SNORD63 binding to an intronic region of the interleukin-4 (IL4) pre-mRNA and helping to recruit the Trf4-Air2-Mtr4 (TRAMP) complex, bringing about degradation of the IL4 mRNA via nuclear exosomes [27]. In another study, a piRNA derived from SNORD75, bound the PIWI proteins PIWIL1 and PIWIL4 and facilitated the exchange of repressive histone marks (H3K27me3) with active ones (H3K4me3). This resulted in an increase in expression of the TRAIL tumor suppressor [19]. The precise mechanisms and the extent to which snoRNAs serve as a source of piRNAs, or miRNAs, (Figure 5b) remain to be determined; however, it is clear that snoRNAs are capable of providing a source for these smaller gene regulatory non-coding RNAs [155].

The functionality of sdRNAs remains an unresolved question, though there are intriguing suggestions of functional roles. For example, SNORD115 was found to regulate the splicing of HTR2C [147] to produce a longer isoform that includes an alternate exon (5b), and to do this in the absence of extensive RNA editing that is otherwise required for long isoform production [148]. SNORD115 was further shown to regulate alternate splicing events in other pre-mRNAs [150], whilst SNORD88C was found to regulate the splicing of FGFR3 [152]. Both SNORD88C and SNORD115 are processed into shorter fragments, and at least some of these fragments overlap the interaction sites associated with splicing regulation; however, the specific involvement of the sdRNA, but not the snoRNA itself, is currently unclear. Accordingly, the mechanism through which splicing is regulated is not fully elucidated; however, it is worth noting that sdRNAs interact with a set of heterogenous ribonucleoproteins (hnRNPs) that are distinct from the interaction partners of the full length snoRNAs and that themselves have been implicated with splicing [147].

### 4.2. SnoRNA-Derived RNAs (sdRNAs)—Biogenesis

The mechanism, or mechanisms, that generate smaller fragments from longer snoRNAs remain little understood; however, it has been demonstrated that at least some sno-RNA-derived miRNAs (such as that derived from ACA45) are DICER-dependent [25]. Additionally, deep sequencing analysis of wildtype and DICER/DGCR8 knockout of ESCs (embryonic stem cells) revealed a difference in the length distribution of H/ACA box snoRNAs-derived fragments, suggesting a widespread involvement of the microprocessor (DICER/DGCR8) complex in a manner reminiscent of miRNA biogenesis [17]. In contrast, however, the production of smaller fragments from C/D box snoRNAs was unaffected by DICER/DGCR8 knockout [17]. Global cross-linking of cellular RNAs to DGCR8 however did reveal extensive snoRNA–DGCR8 interactions, and DGCR8-mediated cleavage of snoRNAs was DICER-independent, suggesting a possibility that one sdRNA biogenesis pathway might involve enzymes other than DICER associating with DGCR8 [156].

It has been reported that the production of sdRNAs increases during non-optimal or stress conditions, suggesting potential roles in stress regulation. Consistent with this, the sdRNAs produced under these conditions are associated with ribosomes, suggesting yet unidentified roles of sdRNAs in translation. This is an intriguing possibility given that translation typically decreases under stress conditions [157,158]. Again, much remains to be uncovered regarding the roles of stress-induced sdRNAs, though it should be noted that tRFs generated under stress conditions have also been linked to the regulation of translation through their competition with mRNAs for ribosome association [159]. This suggests that fragments arising from different ncRNAs under stress conditions facilitate reprogramming of translation [160,161].

In addition to the DICER/DGCR8-associated pathways that one may expect for a small non-coding RNA, recent studies have also implicated the involvement of FUS (fused in sarcoma/translocated in liposarcoma) in the generation of sdRNAs [161,162]. FUS, a nuclear-localized RNA-binding protein, is a component of a heterogeneous nuclear ribonucleoprotein (hnRNPs) complex with established roles in RNA splicing, RNA transport, and DNA repair [163]. Interestingly, RNA immunoprecipitation and high-throughput sequencing has demonstrated that miRNAs and all classes of snoRNAs are immunoprecipitated with FUS [161]. FUS enhances gene silencing by associating with miRNAs and its mRNA targets [164]. Furthermore, upon FUS over-expression, the levels of sdRNAs increased whilst expression of mature snoRNAs was decreased [39,161]. Precursor snoRNA levels were unaffected, suggesting that FUS plays a role in the generation of sdRNAs but not in snoRNA biogenesis. In addition to snoRNAs, tRFs also co-immunoprecipitated with FUS, implying that FUS might be involved in the generation of more than one type of small RNA [161].

## 5. Conclusions

For decades, the known functions of snoRNAs were limited to the post-transcriptional modification of RNAs associated with translation. However, previously unexpected roles for these molecules have been reported; the intricacies of which are still being discovered [128]. In addition to being involved with pre-rRNA cleavage, snoRNAs have been reported to bind directly to mRNA, regulating 3′ mRNA processing and alternative splicing [34]. The biological significance of these non-canonical roles for snoRNAs, such as in alternate splicing, is demonstrated in Prader–Willi Syndrome, a condition characterized, at least in part, by hyperphagia leading to obesity [165]. Another prominent example is shown by the depletion of SNORD50A, which alters the polyadenylation of mRNAs, as it has a role in 3′ mRNA-processing [153].

Various studies suggest that the non-canonical functions of snoRNAs may be facilitated by the metabolically stable fragments they give rise to [42]. Deep sequencing and bioinformatic analysis have revealed that in addition to generating miRNA-like fragments, H/ACA and C/D box snoRNAs produce fragments both smaller (17–19 nt) and larger (>27 nt) than AGO-associated miRNAs [166]. This, coupled with the conserved production of these fragments across species [152], suggests the possibility of functional roles for sdRNAs that are distinct from simply serving as an additional source of miRNA. Though the existence of stable fragments is in and of itself insufficient evidence for function, there are at least suggestions of functional sdRNAs, such as those derived from SNORD115 and SNORD88C, that may be involved in splicing regulation [42,165]. Additional examples are likely to follow. This has parallels to other classes of ncRNA, where the generation of smaller stable fragments is also common [104] and for which examples of biological significance have also been ascribed. Additionally, in parallel to other classes of ncRNA, the expression of sdRNA fragments is often altered in pathological conditions such as cancer [167,168,169], though the extent to which they drive the pathology or merely act as indicators of disease progression remains uncertain.

## 6. Perspectives

The interest in this emerging area of molecular biology is both in the nature of the “new” mechanisms that are regulated by snoRNAs and sdRNAs and in the breadth of genes that are subject to regulation. Are we looking at unexpected roles for a handful of snoRNAs regulating a few dozen genes, or does the work we discuss here represent the first insights into a new frontier for molecular discovery?

At this stage, it is impossible to answer this question; however, there are several factors that at least suggest the possibility that non-canonical functions of snoRNAs could represent a larger tool of gene regulation than is currently appreciated. First, snoRNAs and sdRNAs are abundant, yet frequently overlooked in sequencing data where experimental and bioinformatic tools are poorly developed to either capture or analyze such RNAs. Second, snoRNAs are seemingly capable of multiple interactions, both in terms of the assembly of non-canonical snoRNPs and in their capacity to interact with target transcripts across imperfect binding interfaces. Coupled with the existence of several hundred orphan snRNAs (which are conserved but seemingly devoid of traditional ncRNA targets), these lines of evidence are collectively suggestive of important non-canonical roles.

This has ramifications both in increasing our understanding of molecular and gene regulatory pathways and in the application of non-invasive biomarkers, where snoRNA/sdRNA dysregulation has already been widely reported in cancer but is yet to factor into clinical decision making.

## Data Availability

No new data were created or analyzed in this study. Data sharing is not applicable to this article.

## Abbreviations

Abbreviations	Meaning
ncRNAs	Non-coding RNAs
rRNA	Ribosomal RNA
tRNA	Transfer RNA
miRNA	microRNA
piRNA	PIWI-interacting RNA
siRNA	Small interfering RNA
lncRNA	Long non-coding RNA
paRNAs	Promoter-associated RNAs
SnoRNA	Small nucleolar RNA
sdRNA	snoRNA-derived fragments
snRNA	Small nuclear RNAs
snoRNP	snoRNA–ribonucleoprotein complexes
scaRNAs	Small Cajal body specific RNAs
RISC	RNA-induced silencing complex
RITS	RNA-induced transcriptional silencing1
RTD	Rapid tRNA decay
tRFs	tRNA-derived fragments
ANG	Angiogenin
AGO	Argonaute
tiRNAs	Stress-induced tRNA fragments
tRF-GG	tRF-Gly-GCC
ERV	Endogenous retroviral element
HCC	Hepatocellular carcinoma
CRCs	Colorectal carcinomas
ALDH	Aldehyde dehydrogenase
NSCLC	Non-small cell lung cancer
Htr2c	Serotonin receptor 2c
A–I	Adenosine-to-inosine
FGFR3	Fibroblast growth factor receptor
CPSF	Cleavage and polyadenylation specificity factor
HEK293	Human embryonic kidney 293 cells
ACA45	H/ACA scaRNA15
IL-4	Interleukin-4
TRAMP	Trf4-Air2-Mtr4
FUS	Fused in sarcoma/translocated in liposarcoma
hnRNPs	Heterogeneous nuclear ribonucleoprotein
